# Marine-Derived Astaxanthin: Molecular Mechanisms, Biomedical Applications, and Roles in Stem Cell Biology

**DOI:** 10.3390/md23060235

**Published:** 2025-05-29

**Authors:** Aretha Rambaldi, Francesca Paris, Pasquale Marrazzo, Roberta Costa, Stefano Ratti, Francesco Alviano

**Affiliations:** 1 Cellular Signalling Laboratory, Anatomy Center, Department of Biomedical and Neuromotor Sciences (DIBINEM), University of Bologna, 40126 Bologna, Italy; aretha.rambaldi@studio.unibo.it (A.R.); francesca.paris6@unibo.it (F.P.); r.costa@unibo.it (R.C.); stefano.ratti@unibo.it (S.R.); francesco.alviano@unibo.it (F.A.); 2 Department of Biomolecular Sciences, University of Urbino, 61029 Urbino, Italy

**Keywords:** astaxanthin, stem cells, cytoprotection, carotenoid, marine microalgae, oxidative stress, NF-κB, Nrf2 signaling, inflammation

## Abstract

Astaxanthin (ASX) is a xanthophyll carotenoid mainly derived from marine microalgae such as *Haematococcus pluvialis* and *Chlorella zofingiensis*, as well as the yeast *Phaffia rhodozyma*. Its chemical nature structure, rich in conjugated double bonds, carbonyl, and hydroxyl groups, confers potent antioxidant and anti-inflammatory properties. ASX modulates oxidative stress via the PI3K/Akt-Nrf2 pathway and suppresses NF-κB-mediated inflammatory responses, reducing cytokine levels such as TNF-α, IL-6, and iNOS. ASX exerts dual apoptotic effects, cytoprotective in non-transformed cells and pro-apoptotic in cancer cells through p53 activation. Sustainable extraction techniques, especially supercritical CO_2_, have improved its industrial applicability. Recent findings highlight ASX’s role in stem cell biology, enhancing proliferation, supporting lineage-specific differentiation, and protecting against oxidative and inflammatory damage, which is a crucial issue for regenerative medicine applications. These multifaceted molecular effects support ASX’s therapeutic potential in chronic diseases, including diabetes, cardiovascular pathologies, and cancer. This review outlines ASX’s natural sources, extraction methods, and biological mechanisms, emphasizing its application in oxidative stress- and inflammation-related conditions.

## 1. Introduction

Astaxanthin (ASX), a marine-derived xanthophyll carotenoid, is attracting increasing attention due to its exceptional antioxidant, anti-inflammatory, and cytoprotective properties. These properties are particularly relevant in the context of chronic and degenerative diseases such as cardiovascular disorders, diabetes, and cancer, where oxidative stress and inflammation are known to drive pathogenesis [1,2]. Despite numerous studies highlighting its bioactivity, a comprehensive and mechanistically oriented synthesis of current evidence, particularly linking ASX to molecular pathways involved in redox balance, inflammation, apoptosis, and tissue repair, remains limited in the literature.

This review aims to fill that gap by offering a detailed and updated overview of the chemical structure, natural sources, extraction technologies, and biomedical effects of ASX, with a special focus on its molecular mechanisms of action. In addition to consolidating evidence in classical fields such as metabolic and cardiovascular diseases, we highlight the emerging role of ASX in stem cell biology and regenerative medicine, a perspective that is still underrepresented in current reviews. We also discuss the influence of structural isomerism (cis/trans), formulation strategies, and bioavailability on its pharmacological activity, integrating recent experimental findings.

A comprehensive literature search was performed using PubMed, Scopus, and Web of Science databases. The selection included peer-reviewed articles published between 2004 and 2024, focusing on the biochemical, pharmacological, and therapeutic properties of ASX and its applications in different biological models. Through this integrative approach, we aim to provide researchers and clinicians with a solid framework to guide future translational applications of ASX in medicine.

### 1.1. ASX Structure

**Figure 1:**
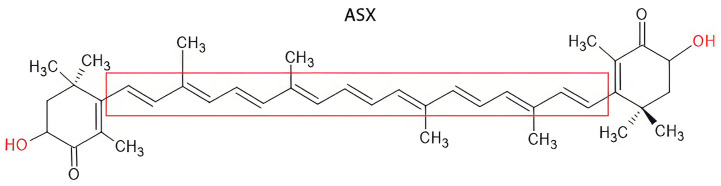
Chemical structure of ASX.

ASX is a lipophilic carotenoid belonging to the tetraterpenoid family. Chemically, it consists of a long-conjugated polyene chain characterized by two carbonyls and two hydroxyl functional groups that play a crucial role in its biological activity (Figure 1) [3].

In particular, the high presence of conjugated double bonds in the principal chain allows for free radical absorption, while the carbonyl group acts as an electron acceptor, contributing to the stabilization of the molecule in the presence of reactive oxygen species (ROS) [4].

The trans-isomer of ASX is characterized by a long, conjugated polyene chain (highlighted in red) flanked by two ionone-type end groups, each containing a hydroxyl (–OH) and a keto (C=O) functional group. These features confer strong antioxidant properties, allowing efficient scavenging of ROS and stabilization of cellular membranes. The *trans* configuration, which predominates in nature, provides enhanced stability and higher biological activity compared to its *cis* counterpart [5].

**Figure 2:**
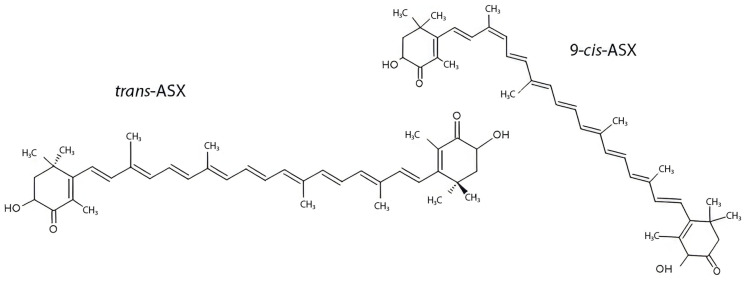
ASX isomerism: trans-ASX vs. 9-cis-ASX structural isomers.

Instead, hydroxyl groups contribute to the amphipathic nature of the molecule, favoring interaction with phospholipid membranes and enhancing bioavailability. Because of the molecule’s chemical structure, ASX can be present in *cis* or *trans* configuration, which can influence the chemical and thermodynamic stability of the system (Figure 2). In nature, *trans*-ASX is the most frequent form, most stable, and biologically active [6].

ASX exists predominantly in the *trans* configuration, which is the most thermodynamically stable and biologically active form. The 9-cis isomer differs in the geometry of one of its double bonds, resulting in distinct spatial orientation and potentially different bioactivities. Both forms contain conjugated double bonds and terminal ionone rings with hydroxyl and keto groups, which are essential for their antioxidant properties. Structural isomerism influences solubility, membrane integration, and interaction with molecular targets, impacting ASX’s pharmacological profile [7].

Due to its lipophilicity, ASX tends to form molecular aggregates in polar solvents. According to the different aggregation patterns, the spectroscopic analysis revealed a blue-shifted aggregation (H-aggregates, 388 nm absorption peak) and red-shifted aggregation (J-aggregation, 565 nm absorption peak). These two different aggregation models show separate behaviors, affecting the stability, photosensitivity, and pharmacological activity of the molecule. H-aggregates adopt a face-to-face structural configuration, whereas J-aggregates exhibit a head-to-tail arrangement. The former, due to shorter intermolecular distances and enhanced π–π interactions, demonstrates greater stability in polar solutions and superior antioxidant activity compared to J-aggregates. In contrast, J-aggregates require a longer aggregation time (approximately one hour), suggesting that extended aggregation periods influence their biological properties [8,9]. The specific chemical properties of ASX are closely linked to its natural sources, such as microorganisms and algae, that can synthesize this molecule.

### 1.2. ASX Sources

ASX molecule occurs in a wide range of species across different taxonomic classes, ranging from bacteria to eukaryotic organisms [10]. While algae are considered the main source for its extraction, ASX can also be derived from plants or marine animals, which can accumulate through the diet [11].

#### 1.2.1. *Haematococcus pluvialis*

One of the most representative ASX producers is *Haematococcus pluvialis*, a microalgae that can grow both in freshwater and aquatic environments characterized by high concentrations of NaCl [12]. It is a unicellular and spheroidal microalga that can exist in a vegetative state or in a quiescent state characterized by ASX accumulation. In the quiescent state, ASX is stored in lipid bodies, where it undergoes esterification, originating mono- and diesters, improving the biological activity [13]. Biokinetic analysis has demonstrated that esterified forms of ASX can influence both thermal stability and bioavailability, demonstrating that a higher esterification level contributes to improved molecular stability [9]. Maximizing the extraction yield requires adherence to the cultivation, harvesting, and extraction protocol. First of all, the choice of culture medium is fundamental because the right temperature (25 ± 5 °C) and low light conditions are mandatory.

One of the critical aspects of the extraction process resides in the rigid structure of the algae cell wall, which could affect the amount of ASX obtained [14]. Many methods of extraction can be performed, including physical (homogenization bead milling, ultrasonication), chemical (organic solvents, supercritical fluid extraction [12], ionic fluid, acid treatment, and nanomaterials) [15], and biological methods (enzyme treatment, germination, milking). Among all extraction methods, supercritical fluids are the most commonly used. Supercritical fluids extraction with CO_2_ can be widely employed not only due to the high-quality standard of the obtained product but also because CO_2_ can be recycled, making the process more sustainable. Supercritical fluids make it possible to extract large amounts of non-polar molecules from the matrix, with a higher yield compared to other chemical extraction methods [15].

Co-solvents such as methanol and ethanol can be employed to further enhance the extraction process and selectivity. The co-solvents act as modulatory agents to improve the solubilization of polar substances, reducing the extraction of compounds that are not desired (Table 1) [16].

#### 1.2.2. *Chlorella Zofigiensis*

Among microalgae sources of ASX, *Chlorella zofingiensis* represents a viable alternative. It is a unicellular microorganism characterized by the presence of chloroplasts that can accumulate chlorophyll, starch, and, interestingly, carotenoids, including ASX. The production of carotenoids as secondary metabolites is an indicator of cellular stress, demonstrating the high adaptative capacity of plants in response to environmental stress [17,18]. Then, optimizing culture conditions (such as stable levels of CO_2_) is essential for regulating ASX accumulation. Correct nutrients and an appropriate environment are critical elements for efficient algae growth and achieving a high extraction yield. This alga can efficiently assimilate mono- and disaccharides from glucose, fructose, or mannose, while other sources of carbon, such as lactose or galactose, are not well absorbed [19]. Different carbon sources can influence ASX yield—for example, a culture medium enriched with glucose or mannose leads to a higher ASX yield (Table 1) [19].

#### 1.2.3. *Phaffia rhodozyma*

*Phaffia rhodozyma* is a red yeast belonging to the class of Basidiomycetes. This yeast undergoes a life cycle characterized by sexual and asexual states, during which ASX and other carotenoids can be accumulated. To increase the amount of ASX accumulated in the microorganism, the nitrogen level of the culture medium must be critically controlled. Moreover, culture temperatures must range between 18 and 22 °C to allow both yeast growth and efficient ASX accumulation (Table 1) [20].

**Table 1:** Comparative overview of ASX production from different biological sources, including extraction conditions, chemical form, and key advantages and disadvantages.

ASX Source	ASX Yield	Chemical Form	Extraction Method	Advantages	Disadvantages
***Haematococcus pluvialis***	207.67–292.70 mg/g DW [12]	Mostly esterified [10]	Supercritical fluid extraction with ethanol [12]	Highest known natural yield; well-characterized cultivation protocols	Expensive cultivation; requires stress conditions to induce accumulation
***Chlorella zofingiensis***	~0.24% of DW (~2.400 μg/g) [17]	Mix of free and esters [17]	Solvent extraction (ethanol, acetone, DMSO, etc.) [17]	Easy to cultivate; high growth rate	Lower yield; co-production with other carotenoids (e.g., lutein)
***Phaffia rhodozyma***	~1294.7 μg/g DW [21]	Free form [22]	Acidic or enzymatic cell wall disruption + solvent [21]	Yeast-based system; suitable for industrial-scale fermentation	Lower yield than microalgae; extraction requires cell disruption

In scientific literature, numerous other natural and synthetic sources of ASX have been identified. However, due to their relatively low ASX content, the extraction process is not economically convenient.

### 1.3. Biological Activities of ASX

Similarly to other carotenoids, ASX exhibits a wide range of biological activities. Based on its mechanism of action, these can be mainly classified into antioxidant, anti-inflammatory, and apoptosis-regulating properties.

#### 1.3.1. ASX as a Modulator of Oxidative Stress via PI3K/Akt-Nrf2 Axis

Oxidative stress and inflammation are closely interconnected, playing a fundamental role in the onset and progression of many chronic diseases. Under stress conditions, excessive production of ROS leads to oxidative damage. ROS are involved in membrane disruption, lipid peroxidation, and DNA damage, impairing cellular functionality [23]. The consequences of ROS exposure depend on the duration, strength of pro-oxidant substance, and context of exposure. When the cell undergoes prolonged stress conditions, p53 and p21 proteins are translated and lead to cell cycle arrest [24]. Hydroxyl radical (OH•), generated during oxidative metabolism, reacts with DNA bases, causing structural modifications in genetic material and mutation [25]. Such genetic alteration can lead to errors during protein transcription, triggering body homeostasis and leading to the development of various pathologies.

The principal ROS sources in cells are mitochondria, NADPH oxidase, and cytochrome p450. Above all, mitochondrial dysfunction is related to an increased production of O2•− via the electron transport chain during the cell respiratory process. Physiologically, the human body can contrast oxidative stress by producing antioxidant enzymes. The principal antioxidant enzymes are glutathione peroxidases (GPX), catalase (CAT), and superoxide dismutase (SOD) family enzymes [26].

**Figure 3:**
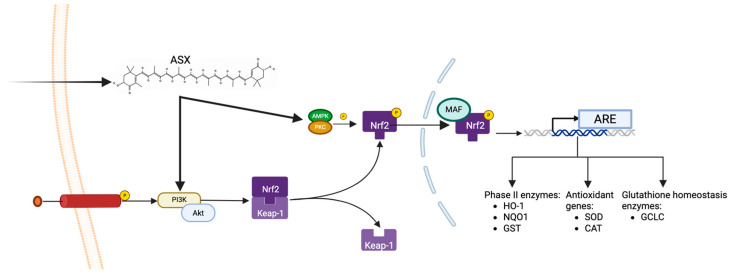
ASX-mediated activation of the PI3K/Akt-Nrf2 antioxidant pathway.

ASX exerts its antioxidant activity through different mechanisms of action. One of its key molecular targets is the PI3K/Akt signaling pathway, which is fundamental for cell survival, metabolism, and oxidative stress response. This pathway is involved in radical scavenging through the Nrf2/ARE axis. Dysregulation of the PI3K/Akt pathway is implicated in human malignancies, including cancer, Alzheimer’s, cardiovascular diseases, diabetes mellitus, and neurological diseases [27]. In these situations, the organism undergoes chronic inflammation and oxidative stress, which leads to abnormal activity of the pathway. Upstream of the activation of this pathway, cytokines, growth factors, receptor tyrosine kinase (RTK), and G-protein coupled receptors act in response to their binding with specific ligands or adhesion molecules [28]. ASX increases the activity of the PI3K/Akt pathway, which is directly related to the activity of Nfr2, a transcription factor responsible for the constitutive and inducible expression of ARE-dependent genes involved in oxidative stress response. Physiologically, Nrf2 is associated with Keap1, but after oxidative stress, a conformational change of Keap1 induces its dissociation from Nrf2, which is phosphorylated by protein kinase C (PKC) and AMP-activated protein kinase (AMPK). Phosphorylation by AMPK is essential for Nrf2 nuclear translocation to increase overall cell survival [29]. Indeed, upon Nrf2 phosphorylation, it translocates through the nuclear membrane and is linked to MAF transcription factors, then the Nrf2-MAF complex binds to ARE elements, leading to antioxidant gene transcription. The radical scavenging activity can be performed through the transcription and synthesis of phase II enzymes heme oxygenase-1 (HO-1), NAD(P)H quinone dehydrogenase 1 (NQO1) and glutathione S-transferases (GST), antioxidant enzymes and enzymes involved in glutathione homeostasis glutamate-cysteine ligase (GCLC) and glutathione reductase (GSR) (Figure 3) [30].

ASX activates the PI3K/Akt signaling cascade, promoting the phosphorylation and nuclear translocation of Nrf2. After dissociation from Keap1, Nrf2 is stabilized and further phosphorylated by AMPK and PKC. Nrf2 heterodimerizes with MAF proteins in the nucleus, and this heterodimer binds to antioxidant response elements (ARE) in the DNA, inducing the transcription of phase II detoxifying enzymes (e.g., HO-1, NQO1, GST), classical antioxidant enzymes (e.g., SOD, CAT), and glutathione-homeostasis enzymes (e.g., GCLC), thereby promoting cellular survival.

#### 1.3.2. Anti-Inflammatory Properties of ASX: A Lens on NF-κB Pathway

**Figure 4:**
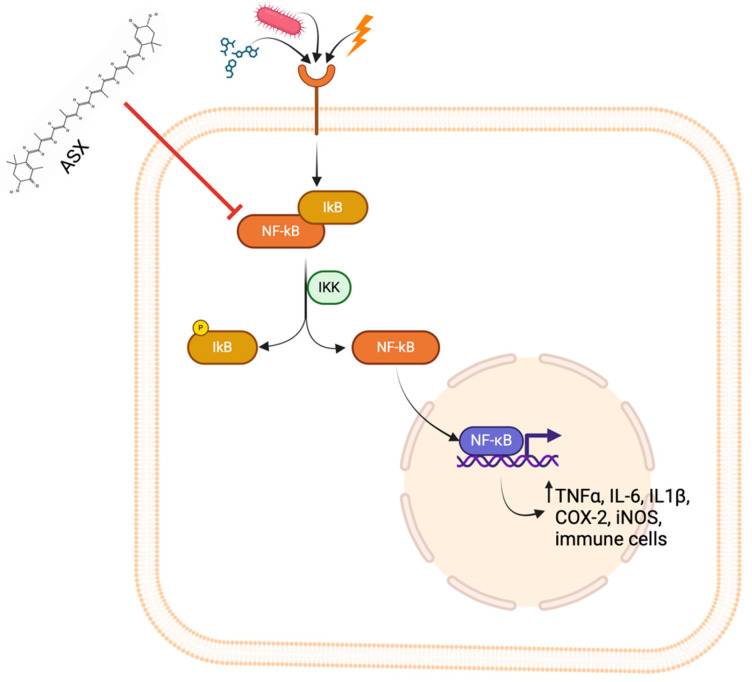
Inhibitory effect of ASX on the NF-κB signaling pathway.

Beyond its role in oxidative stress modulation, ASX exhibits significant anti-inflammatory effects. Oxidative stress induces inflammation, considering free radicals’ role in releasing inflammatory markers. Inflammation is the first responsive measure that the body activates to defend itself against injuries that derive from physical, chemical, and biological stimuli [31]. Inflammation reaction leads to microcirculatory changes, including vascular permeability alteration, leukocyte recruitment, and accumulation and release of pro-inflammatory mediators [32]. The inflammatory cascade is activated when foreign agents are recognized by pattern recognition receptors (PRRs). These receptors can recognize both damage-associated molecular patterns (DAMPs) and pathogen-associated molecular patterns (PAMPs), triggering intracellular signaling pathways that can activate inflammatory response [33]. The nuclear factor kappa B (NF-κB) pathway represents one of the principal signaling cascades involved in inflammation. The activation of this pathway is associated with augmented production of pro-inflammatory molecules, immune infiltration, and regulation of antioxidant enzyme expression. In response to stimulation by cytokines, microorganisms, oxidative stress, and chemicals, PRRs initiate the activation of the NF-κB pathway. In resting conditions, NF-κB is situated in the cytoplasm bound to the inhibitory protein I IkB. After exposure to a pro-inflammatory stimulus, IkB is phosphorylated and ubiquitinated and is separated from NF-κB, which is translocated into the nucleus. Once NF-κB is inside the nucleus, it binds DNA sequences, leading to the transcription of genes encoding proteins with pro-inflammatory activity, such as interleukin-6 (IL-6), cyclooxygenase-2 (COX-2), inducible nitric oxide synthase (iNOS) and tumor necrosis factor-α (TNF-α) [34]. ASX exerts its anti-inflammatory activity by inhibiting the NF-κB pathway, leading to a reduced amount of pro-inflammatory cytokines. A study conducted by Cai et al. [32] demonstrated that pre-treatment with ASX before stimulation by lipopolysaccharide (LPS) inhibited the degradation of IkB, suppressing the activation of the NF-κB pathway in RAW264.7 macrophages and reducing the production of pro-inflammatory cytokines, such as TNF-α, IL-6, and iNOS (Figure 4).

Upon stimulation by pro-inflammatory signals such as PAMPs, cytokines, or oxidative stress molecules, the NF-κB pathway is activated through phosphorylation of IκB by the IKK complex, leading to IκB degradation and translocation of NF-κB into the nucleus, where NF-κB promotes transcription of pro-inflammatory mediators, including TNF-α, IL-6, IL-1β, COX-2, and iNOS. ASX inhibits this pathway by preventing IκB degradation and NF-κB nuclear translocation, thereby reducing the inflammatory response and immune cell activation.

#### 1.3.3. Role of ASX on Apoptosis

Apoptosis is a strictly regulated cellular mechanism necessary to maintain cellular homeostasis. By eliminating damaged, dead, or dysfunctional cells, apoptosis contributes to tissue renewal, avoiding the accumulation of dangerous stimuli capable of inducing tissue damage and functional deterioration [35]. The two main apoptosis pathways, extrinsic and intrinsic pathways, converge on the activation of caspases-3 and -7, which play a pivotal role in apoptosome formation and the execution of programmed cell death [36]. One of the master regulators of apoptosis is the p53 protein (encoded by the TP53 gene), whose expression is increased after cytokine deprivation, DNA damage, or oxidative stress. p53 targets specific genes, resulting in the inhibition of anti-apoptotic proteins BCL-2 and improving the cytochrome C release [37].

**Figure 5:**
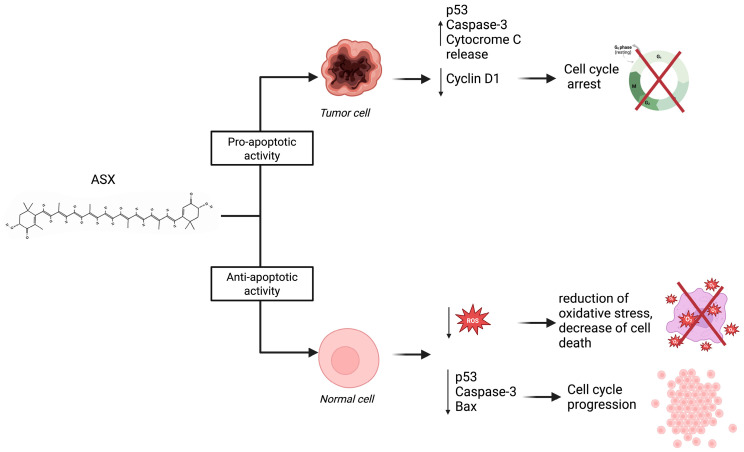
The dual role of ASX in modulating apoptosis depending on cell type.

Notably, ASX has been shown to exert both pro- and anti-apoptotic effects, according to cellular context. As for anti-apoptotic activity, a study conducted by Bi et al. [38] on blastocysts—derived through somatic cell nuclear transfer (SCNT)—using fibroblasts—derived from newborn ear calf tissue—demonstrated that the group treated with ASX showed downregulation in pro-apoptotic gene expression compared with the control group. Tp53, Bax, and Caspase-3 genes are downregulated, while the expression of anti-apoptotic gene Bcl2l1 is increased [38]. Moreover, ASX shows anti-apoptotic activity in granulosa cells (GS) exposed to hydrogen peroxide (H_2_O_2_), which is responsible for cell apoptosis. After ASX pre-treatment, cells showed a significant reduction in apoptosis rate compared with the control group, which did not receive pre-treatment [39]. In contrast, in cases of excessive cell proliferation, such as in tumors, ASX has demonstrated a pro-apoptotic effect, reducing cancer cell proliferation and tumor growth. SKBR3 [40] and MCF-7 [41] cell lines of breast tumors incubated with defined concentrations of ASX showed an increase in apoptotic cells compared with untreated control cells. Particularly, studies on MCF-7 cells demonstrated that ASX increases the expression of p53 and caspase-3, leading to cell cycle arrest and apoptosis. Moreover, ASX inhibits antagonists of p53 such as MDM2, increasing its pro-apoptotic effect. ASX demonstrated pro-apoptotic activity on colorectal cells (LS-180 cell line), evaluating apoptosis-related genes. Indeed, Caspase-3 and Bax were downregulated after administration of 100µM of ASX compared with the control group. On the other side, ASX can inhibit the expression of anti-apoptotic gene Bcl-2 [42]. As a result, ASX alters the progression of the cell cycle at the G0/G1 phase, increasing p53 expression and reducing cyclin D1 expression, a molecule directly involved in tumoral cell proliferation (Figure 5) [43].

ASX exerts pro-apoptotic effects in tumor cells by increasing p53 expression, activating caspase-3, and promoting cytochrome C release while downregulating Cyclin D1 expression, resulting in cell cycle arrest. In contrast, ASX shows anti-apoptotic activity in normal cells by reducing intracellular ROS levels and downregulating pro-apoptotic factors such as p53, caspase-3, and Bax. This leads to decreased oxidative stress, reduced cell death, and promotion of normal cell proliferation. These differential effects highlight ASX’s potential as a selective therapeutic agent in cancer treatment while preserving healthy tissue.

#### 1.3.4. Immunomodulatory Properties of ASX

The immune response is a highly coordinated defense mechanism that enables the host to detect and eliminate pathogenic threats while maintaining tissue homeostasis. It is traditionally divided into two branches: the innate and the adaptive immune systems. The innate immune response constitutes the first barrier against invading microorganisms. It includes anatomical and physiological components such as the skin, mucosal epithelia, and associated secretions, which act to prevent pathogen entry [44].

Innate immune cells, including macrophages, neutrophils, dendritic cells, mast cells, and natural killer (NK) cells, are recruited to the site of infection. These cells mediate the initial clearance of pathogens through phagocytosis, release of cytotoxic granules, and induction of programmed cell death (apoptosis). If the innate mechanisms are insufficient to eradicate the pathogen, the adaptive immune response is initiated. This involves the activation of B lymphocytes and T lymphocytes, characterized by highly specific receptors generated through somatic recombination. These lymphocytes originate and undergo maturation in primary lymphoid organs, the bone marrow and thymus, before migrating to secondary lymphoid tissues, such as the lymph nodes and spleen. There, they are activated by antigen-presenting cells (APCs) that display processed antigens via major histocompatibility complex (MHC) molecules [45].

ASX has gained increasing attention for its immunomodulatory properties. Beyond its well-established antioxidant, anti-inflammatory, and anti-apoptotic activities, astaxanthin has been shown to influence both innate and adaptive immune responses [46]. It enhances lymphoproliferation, NK cells’ cytotoxic activity, and pro-inflammatory cytokines. This leads to an increase in T and B cells, improving immune response. A study conducted by Park et al. on university students showed ASX augmented cell proliferation and increased NK cytotoxic activity, while decreased pro-inflammatory markers such as 8-hydroxy-2′-deoxyguanosine (8-OHdG) and C-reactive protein (PCR) [47].

## 2. Therapeutic Potential of ASX in Human Diseases

The interplay between oxidative damage, chronic inflammation, and abnormal cell signaling can push toward the development of a wide range of pathological conditions. Considering its involvement in key cellular mechanisms—described in the below sections—such as inflammation, oxidative stress, and apoptosis, ASX can play a relevant role in maintaining cellular homeostasis and overall physiological balance.

### 2.1. ASX in Cardiovascular Diseases

Cardiovascular diseases are one of the most common causes of death in the world, with unhealthy dietary habits and sedentary lifestyles as key triggering factors [48]. The oxidation of lipids, particularly low-density lipoprotein (LDL), promotes their accumulation within the vascular wall, increasing the risk of developing atherosclerosis and endothelial dysfunction. Oxidative imbalance can alter blood rheology, inducing an increase in platelet aggregation and vasoconstriction [1]. ASX has been shown to reduce LDL oxidation [49] and radical production while increasing the activity of antioxidant enzymes, e.g., SOD and CAT. Additionally, ASX activates the Nrf-2/HO-1 pathway, inducing antioxidant response and controlling blood integrity. ASX stimulates the NO release that directly acts on blood vessels by inducing vasodilatation [50]. Regarding inflammatory components, oxidized LDL (oxLDL) represents an important inflammation marker. Macrophages bind oxLDL by activating specific receptors such as LOX-1 and CD36, releasing pro-inflammatory cytokines, chemokines, and metalloproteases (MMPs). This leads to extracellular matrix degradation and the generation of atherosclerotic plaques [51]. Linking to the NF-κB pathway, upon stimulation by pro-inflammatory molecules such as ROS and lipopolysaccharides, I*κ*B proteins are phosphorylated by IKK protein. Nuclear NF-κB promotes pro-inflammatory gene transcription. ASX exerts anti-inflammatory activity by inhibiting IKK activity, reducing nuclear translocation and pro-inflammatory mediators’ production, such as NO, tumor necrosis factor-α (TNF-α), interleukin-1-beta (IL-1β) and cyclooxygenase-2 (COX-2) [52].

In a pilot study conducted by Kato et al., three months of ASX administration significantly reduced systemic oxidative stress (measured by dROM parameter) and improved left ventricular ejection fraction (LVEF) and exercise tolerance (6MWD). These modifications can be explained by the reduction in oxidative stress by ASX [53]. As for myocardial fibrosis, Zhang et al. conducted a study on mice subjected to constriction of the left common carotid arteries. This study revealed that ASX administration mitigates cardiac fibrosis by suppressing TGF-β1 expression, a key profibrotic cytokine involved in pathological cardiac remodeling [54].

### 2.2. ASX in Diabetes Mellitus

Diabetes mellitus is a chronic metabolic disease that affects more than 530 million people worldwide, with prevalence rates steadily increasing. Regardless of etiology, both type 1 and type 2 diabetes share common pathogenic mechanisms involving oxidative stress and chronic inflammation caused by ROS and RNS [55]. ROS, which originates from the mitochondrial electron transport chain (ETC), endoplasmic reticulum (ER), and peroxisome, can disrupt B-cell insulin secretion, resulting in chronic hyperglycemia. Prolonged elevated blood glucose levels lead to various clinical complications, including retinopathy, nephropathy, cardiovascular diseases, and chronic low-grade inflammation. In this context, the activation of the NF-κB pathway plays a crucial role in amplifying the inflammatory cascade, releasing interleukin-6 (IL-6), TNF-α, and IL-1β [56].

ASX has been reported to exert multiple protective effects in diabetes. Studies conducted on animal models revealed that, according to the diabetes stage of progression, ASX can modulate several cellular pathways. In the early stage of development, ASX improves glucose metabolism and insulin sensitivity, ameliorating antioxidant enzyme secretion while reducing insulin resistance and blood glucose levels [57]. ASX enhances insulin sensitivity by directly acting on the promotion of autophosphorylation of insulin receptor substrate 1/2 (IRS-1/2), which is directly connected with PI3K/Akt axis and Nrf2/ARE activity, regulating antioxidant enzyme production. Moreover, this antioxidant cascade is associated with an augmented translocation of glucose transporter 4 (GLUT4) to the cell membrane, inducing glucose uptake [58]. At the stage of overt diabetes, ASX has been shown to mitigate hyperglycemia, decrease lipid peroxidation, and attenuate both oxidative stress and systemic inflammation. A study conducted by Rizzardi et al. demonstrated the capacity of ASX to reduce lipid peroxidation both in normal and stressed conditions under tert-butyl hydroperoxide (TBH) administration [59]. Moreover, ASX modulates the activity proliferator-activator receptors γ (PPARγ), a master regulator of adipogenesis, inflammation, and insulin sensitivity, and the primary molecular target of the antidiabetic drug thiazolidinedione (TZD). Specifically, ASX modulates the interaction between PPARγ and its coactivators, including transcription intermediary factor 2 (TIF2) and steroid receptor coactivator-1 (SRC-1). The PPARγ modulating activity is particularly relevant in the context of diabetes. Limitation of excessive adipose tissue accumulation and enhanced macrophage anti-inflammatory response, ASX may improve insulin sensitivity and reduce chronic hyperglycemia [60].

In addition, ASX has shown the ability to counteract multiple diabetes-related complications, such as retinopathy, nephropathy, and neuropathy, through a variety of protective mechanisms. Regarding diabetic retinopathy, ASX has been reported to attenuate high glucose-induced lipid peroxidation, downregulate mRNA expression of VEGF and metalloproteinases-2 (MMP2), and suppress cell proliferation. Furthermore, ASX reduces the activation of the NF-κB pathway and aldose reductase activity while enhancing the expression of antioxidant enzymes, thereby providing retinal protection against oxidative stress [61,62]. Diabetic nephropathy, one of the most common complications of diabetes, arises from glomerular and tubular lesions caused by prolonged hyperglycemia and inflammation. Activation of the NF-κB pathway promotes an overproduction of extracellular matrix components (ECM), causing renal fibrosis. Chronic ASX administration has been shown to mitigate hyperglycemia-induced albuminuria and significantly reduce plasmatic levels of urea, creatinine, and uric acid, which are directly related to renal damage. Additionally, ASX attenuates kidney structural damage, including glomerular hypertrophy and fibrosis [63,64]. Beyond renal and retinal protection, ASX also exerts beneficial activity on diabetic neuropathy and hippocampal-based cognitive deficits. Neuropathy in diabetes is primarily caused by the activation of NF-κB, MAPK pathway, and TGF-β, which promote inflammation, apoptosis, and neurovascular impairment. ASX modulates these mechanisms through suppression of NF-κB, decreasing the expression of pro-inflammatory cytokines TNF-α, IL-6, and IL-1β. At the same time, ASX enhances the PI3K/Akt pathway, increasing neuronal cell survival [65].

### 2.3. ASX in Cancer

ASX has emerged as a promising agent in oncology due to its diverse anti-tumor mechanisms and its capacity to enhance the efficacy of standard therapies while modulating immune responses and preserving tissue integrity.

In hepatocellular carcinoma (HCC), ASX has shown the ability to potentiate the effects of sorafenib, a first-line treatment with limited efficacy in many patients. When combined with subclinical doses of sorafenib, ASX increased tumor inhibition to 76.5%, outperforming even the clinical dose of sorafenib alone. This synergy was mediated through inhibition of the JAK2/STAT3 pathway, reduction in intratumoral hypoxia, and promotion of apoptosis by modulating Bax/Bcl-2 ratios and caspase-3 activation [66]. Additionally, ASX enhanced the infiltration of CD8^+^ T lymphocytes and reprogrammed tumor-associated macrophages from the M2 to M1 phenotype via activation of the CXCL9/CXCR3 axis, suggesting an immunomodulatory role alongside its cytotoxicity [67]. In prostate cancer, ASX exerted strong anti-proliferative and anti-invasive effects on DU145 and PC-3 cell lines, primarily through STAT3 inhibition. [68]. Synergistic effects were also observed with cisplatin [69,70], while in vivo, high-dose ASX (100 mg/kg) inhibited PC-3 tumor growth in xenograft models [71]. In glioblastoma models, ASX and its derivative adonixanthin demonstrated the ability to cross the blood–brain barrier and exerted cytotoxic effects on GL261 and U251MG cells, significantly reducing tumor proliferation and migration in vivo at oral doses of 10–30 mg/kg [72]. The translational potential of ASX is further supported by its tolerability and capacity to alleviate chemotherapy-induced tissue damage. Notably, its antioxidant and immunomodulatory effects contribute to reduced toxicity, enhanced mucosal immunity, and preservation of gut microbiota homeostasis during therapy [73].

Collectively, these data position ASX as a compelling adjuvant in cancer treatment, capable of augmenting the efficacy of molecular therapies, enhancing immune competence, and minimizing treatment-associated side effects. Further clinical trials are needed to confirm its safety and standardize therapeutic regimens for translational application across tumor types.

### 2.4. ASX in Neurological Diseases

ASX has emerged as a promising neuroprotective compound due to its ability to cross the blood–brain barrier and its strong antioxidant and anti-inflammatory properties. These characteristics render ASX particularly relevant for neurodegenerative diseases, where oxidative stress and chronic inflammation contribute to neuronal dysfunction and death.

Preclinical studies have consistently demonstrated that ASX can improve cognitive performance and mitigate neuropathological hallmarks in models of Alzheimer’s disease (AD). Its neuroprotective effects are mediated through the reduction in oxidative stress, inhibition of microglial activation, and suppression of pro-inflammatory cytokines. Furthermore, ASX downregulates apoptotic pathways and upregulates neurotrophic signaling, such as the PI3K/Akt and ERK pathways, contributing to neuronal survival and synaptic plasticity [74]

In models of Parkinson’s disease (PD), ASX attenuates dopaminergic neuronal loss, improves mitochondrial function, and reduces α-synuclein aggregation, ultimately leading to improved motor outcomes [75]. Importantly, ASX has also shown efficacy in experimental models of ischemic stroke, where it decreases infarct volume, preserves blood–brain barrier integrity, and reduces inflammatory infiltration and oxidative burden [76].

Beyond specific disease models, ASX has demonstrated the potential to modulate the gut–brain axis by attenuating systemic inflammation and reducing neuroinflammation triggered by peripheral metabolic dysfunctions. These pleiotropic actions make ASX a promising candidate not only for preventive strategies but also as an adjuvant in therapeutic approaches targeting complex neurological disorders.

### 2.5. ASX in Gastrointestinal Diseases

The gastrointestinal tract is frequently exposed to oxidative and inflammatory insults, which are central drivers of tissue damage in a variety of pathological conditions. ASX has shown beneficial effects in multiple gastrointestinal (GI) disorders due to its capacity to scavenge ROS, inhibit pro-inflammatory signaling cascades, and preserve epithelial barrier function.

In the context of inflammatory bowel disease (IBD), ASX supplementation has been shown to reduce colonic inflammation by downregulating cytokines such as TNF-α, IL-1β, and IL-6, while enhancing endogenous antioxidant defenses including superoxide dismutase and glutathione peroxidase. These effects translate into histological improvement and reduced disease activity in experimental models of colitis [77]. Additionally, ASX has been reported to promote tight junction protein expression, thereby maintaining epithelial integrity and limiting intestinal permeability. In gastric pathologies, ASX protects against mucosal injury induced by non-steroidal anti-inflammatory drugs (NSAIDs) and *Helicobacter pylori* infection. It exerts anti-ulcer effects by decreasing oxidative markers, suppressing NF-κB signaling, and facilitating mucosal repair [78].

Recent findings also suggest that ASX may play a preventive role in gastrointestinal carcinogenesis. Through its capacity to reduce oxidative DNA damage, inhibit aberrant cell proliferation, and induce apoptosis in tumor cells, ASX demonstrates chemopreventive potential against colorectal and gastric cancers. These effects are supported by mechanistic studies showing ASX-mediated modulation of key pathways such as JAK/STAT, Wnt/β-catenin, and PI3K/Akt [77].

Collectively, these findings indicate that ASX acts as a multifaceted modulator of gastrointestinal health, capable of exerting both protective and therapeutic effects in chronic inflammatory and neoplastic conditions.

## 3. ASX in Stem Cell Biology

In recent years, stem cell-based therapies have emerged as a cornerstone of regenerative medicine. However, major limitations such as poor survival, limited engraftment, and reduced functional integration of transplanted cells continue to hinder their full clinical success. In this context, the antioxidant and anti-inflammatory properties of ASX offer promising opportunities to enhance stem cell viability and therapeutic performance under stress conditions (Table 2).

### 3.1. Effects of ASX on Stem Cell Proliferation and Stemness

ASX, thanks to its antioxidant properties, has emerged as an innovative modulatory molecule in stem cell biology. Multiple studies indicate that ASX enhances the viability and proliferative capacity of diverse stem cell types under both physiological and stress conditions. For example, ASX treatment significantly increases the proliferation and colony-forming efficiency of neural progenitor cells, activating PI3K/Akt and MEK/ERK signaling pathways and upregulating stemness-related transcription factors [79]. In embryonic neural stem cells, ASX not only boosts self-renewal but also elevates the expression of core pluripotency genes (OCT4, SOX2, Nanog) and cell-cycle regulators (e.g., CDK1/2), thereby “improving stem cell potency” in vitro [80].

Consistently, in mesenchymal stromal/stem cells (MSC) cultures, ASX promotes robust expansion: cells supplemented with ASX-loaded polymeric micelles showed ~26% higher MSC proliferation over one week compared to control. Notably, the ASX pro-growth effect extends to three-dimensional tissue-engineered constructs. MSCs encapsulated in a gelatin-methacryloyl hydrogel proliferate more when ASX is incorporated, highlighting its utility for biomaterial-based regenerative scaffolds [49]. These convergent findings demonstrate that ASX can stimulate stem cell proliferation and survival, likely by activating pro-survival signaling and counteracting oxidative apoptotic triggers, without compromising the fundamental characteristics of stemness.

### 3.2. Enhancement in Multilineage Differentiation by ASX

Importantly, ASX also enhances the differentiation potential of stem cells across multiple lineages. Kim et al. observed that ASX-treated neural stem cells exhibited a greater capacity to differentiate into mesodermal lineages, evidenced by enhanced osteogenic and adipogenic differentiation, demonstrated by upregulation of osteogenic protein markers (osteonectin, osteopontin) and adipogenic enzymes (PPARγ, lipoprotein lipase) [80]. Similarly, in MSCs, ASX augments multilineage differentiation. Low-dose ASX significantly boosts adipocyte, chondrocyte, and osteoblast formation from MSCs (with one study reporting increases of 52%, 106%, and 182% in adipogenesis, chondrogenesis, and osteogenesis, respectively, compared to untreated cells). Consistent with these outcomes, ASX-treated bone marrow MSCs show augmented mineralized matrix deposition after osteogenic induction, underlined by metabolic shifts favoring osteogenesis. Metabolomic analysis revealed ASX alters key metabolites and pathways (e.g., increasing L-tyrosine and pantothenate-CoA biosynthesis) that facilitate osteogenic differentiation, confirming a pro-differentiation effect acting at the metabolic level [81].

The differentiation-supportive effects of ASX are not limited to mesodermal lineages. In fact, in a neural context, it has been shown to promote the differentiation of human adipose-derived stem cells (ASCs or adipose-derived-MSCs) into oligodendrocyte precursor cells with treated cells expressing high levels of oligodendroglial markers and negligible astroglial markers [82], suggesting a directed maturation towards the oligodendrocyte fate (with implications for remyelination therapies in diseases like multiple sclerosis).

Together, these findings illustrate that ASX does not impede stem cell differentiation. On the contrary, it can potentiate the generation of specialized progeny from stem cells, likely by creating a more permissive intracellular environment for lineage-specific gene expression or by mitigating oxidative and inflammatory hindrances to the differentiation process.

### 3.3. Cytoprotection Against Oxidative Stress and Apoptosis

A central feature of ASX’s role on stem cells is its mitigation of oxidative stress and associated apoptosis, which is critical in both in vitro expansion and in vivo regenerative settings. Mesenchymal stem cells are highly susceptible to oxidative damage [83] (e.g., during ischemic transplantation or exposure to cytotoxic agents), and ASX confers significant cytoprotection in such scenarios. Pre-treatment of adipose-derived MSCs with ASX prior to hydrogen peroxide exposure dramatically reduces ROS levels and apoptotic cell death. Mechanistically, ASX activates the endogenous antioxidant defense in these cells by upregulating the transcription factor Nrf2, which in turn elevates downstream antioxidative enzymes like HO-1 and NQO1 [84]. This Nrf2-mediated response augments the cellular ability to neutralize free radicals, explaining the observed preservation of viability. In parallel, ASX shields stem cell mitochondria from oxidative injury. ASX maintains mitochondrial membrane potential and prevents the activation of the intrinsic apoptosis pathway (inhibiting Bax/Caspase-3 signaling), as demonstrated in oxidatively stressed ASCs where ASX rescued mitochondrial function and cell survival. Notably, blocking Nrf2 signaling abolishes these protective effects, confirming that cytoprotection conferred by ASX is largely orchestrated through the Nrf2-ARE pathway [85]. By fortifying stem cells against oxidative stress-induced apoptosis, ASX effectively increases their stress resistance, an invaluable property for cells facing harsh microenvironments, for example, after transplantation or in degenerative disease states.

### 3.4. Anti-Inflammatory Effects on Stem Cells

ASX also exerts anti-inflammatory effects on stem cells, which further enhances their survival and functional integration in pathological models. In metabolic stress conditions, where human bone marrow MSCs were exposed to high levels of saturated fatty acid palmitate, ASX prevented MSC apoptosis and markedly attenuated the palmitate-induced inflammatory response. Palmitate normally triggered elevated secretion of pro-inflammatory cytokines such as IL-6 and monocyte chemoattractant protein-1 (MCP-1) from MSCs, as well as the stress kinase activation (p38 MAPK, ERK) and NF-κB signaling that drives inflammation [86]. ASX treatment blunted these effects, bringing cytokine levels down and modulating kinase/NF-κB activity, thereby preserving a healthier, less inflamed state in the stem cells. Likewise, in ASCs subjected to oxidative stress (mimicking the post-graft environment), ASX suppressed the upregulation of IL-6 and TNF-α that would otherwise occur, correlating with its activation of Nrf2 and inhibition of NF-κB–linked apoptotic signaling. By dampening inflammatory cytokine release and signaling, ASX not only protects the stem cells themselves but could also ameliorate the local microenvironment, as excessive MSC-derived IL-6 or MCP-1 can be detrimental to tissue regeneration. Additionally, by preserving extracellular matrix production (e.g., collagen synthesis) in stressed stem cells [85], ASX helps maintain the structural niche that supports regenerative healing.

In conclusion, the anti-inflammatory and anti-apoptotic effects of ASX reinforce each other to ensure stem cells remain viable, quiescently proliferative, and functionally competent even when experiencing oxidative/inflammatory challenges.

### 3.5. Translational Applications in Regenerative Medicine

The multifaceted benefits of ASX on stem cell viability, differentiation, and stress resilience can translate into significant advantages for regenerative medicine applications. Enhancing the survival of transplanted stem cells is a major goal in cell therapy, and ASX-based preconditioning strategies are actively being explored. Researchers have proposed treating stem cells with ASX prior to transplantation into ischemic or otherwise hostile tissues to improve engraftment outcomes [84]. The rationale is supported by evidence that ASX-preconditioned MSCs better withstand post-transplant oxidative stress, leading to higher cell retention and therapeutic efficacy. In the context of autologous fat grafting, where the retention of grafted adipose tissue depends largely on resident adipose-derived stem cells, ASX shows promise as a supplement to improve graft survival. In a recent study mimicking fat graft conditions, ASX preserved ASC function in a hypoxic, oxidatively stressed milieu and maintained their adipogenic potential, resulting in more robust fat tissue formation [85]. This suggests ASX could be used to augment cell-based soft tissue reconstruction techniques. ASX’s ability to promote oligodendroglial differentiation of MSCs also opens avenues in treating neurodegenerative disorders: ASX-treated stem cells might be exploited for enhancing remyelination in neurodegenerative diseases or protecting grafted neural stem cells in stroke and spinal cord injury models. Furthermore, the integration of ASX into biomaterial scaffolds or delivery systems (e.g., slow-release micelles or hydrogels) provides a means to locally enrich the regenerative niche with this bioactive antioxidant [49,87]. Such approaches have already demonstrated accelerated tissue repair and stem cell growth in situ. Overall, the body of evidence converges to show that ASX empowers stem cells by enhancing their proliferation and differentiation under healthy conditions while imparting robust protection against oxidative and inflammatory stress under pathological conditions. These dual actions, boosting regenerative performance and safeguarding cells in adverse environments, underscore ASX’s potential as a supportive therapeutic in stem cell-based regenerative medicine, warranting further investigation and translation into clinical strategies.

**Table 2:** Summary of ASX effects on stem cells: mechanisms and potential applications.

Experimental Model/Condition	Main Effect of ASX	Key Mechanisms	Potential Application	References
Neural stem/progenitor cells	Increased proliferation, stemness, and oligodendroglial differentiation	Activation of PI3K/Akt and MEK/ERK pathways; upregulation of OCT4, SOX2, Nanog	Neuroregeneration, remyelination	[79,80,82]
MSCs (2D/3D culture)	Enhanced viability, proliferation, and multilineage differentiation	Nrf2 pathway activation; metabolic shift; PPARγ induction	Musculoskeletal repair, tissue engineering	[49,80,81]
MSCs/ASCs under oxidative/metabolic stress	Reduction in ROS, apoptosis, and inflammatory cytokines	Activation of Nrf2/ARE pathway; inhibition of NF-κB and MAPK signaling	Cell preconditioning, fat grafting	[84,85,86]
MSCs in biomaterial systems	Improved cell retention and local tissue regeneration	Local modulation of oxidative microenvironment	Smart scaffolds, regenerative delivery systems	[49,87]

## 4. Conclusions

Growing evidence highlights ASX’s multifunctional role as a bioactive compound capable of targeting key molecular pathways involved in oxidative stress, inflammation, apoptosis, and tissue regeneration. Its ability to modulate redox-sensitive transcription factors such as Nrf2 and NF-κB provides a mechanistic basis for its cytoprotective, anti-inflammatory, and anti-tumor actions. Notably, ASX exhibits context-dependent regulation of apoptosis, protecting healthy cells while promoting programmed cell death in transformed or malignant cells. This dual action represents a significant therapeutic advantage in complex pathological conditions. Furthermore, ASX’s emerging role in stem cell biology, enhancing proliferation, survival, and differentiation while mitigating oxidative damage, positions it as a promising adjuvant in regenerative medicine. Combined with its excellent safety profile and natural origin, these properties support ASX’s potential for clinical translation. Despite the growing body of evidence supporting the biomedical potential of ASX, several questions remain to be addressed. Future studies should focus on elucidating the precise molecular interactions between ASX and intracellular targets, particularly in the context of redox-sensitive transcription factors, epigenetic regulators, and mitochondrial dynamics. Moreover, standardized protocols for ASX formulation, delivery, and dosage are needed to improve reproducibility across preclinical studies and facilitate clinical translation. In the field of regenerative medicine, further investigations are warranted to evaluate the long-term effects of ASX on stem cell function in vivo, its integration into biomaterial scaffolds, and its potential synergistic use with other bioactive compounds or genetic modulation strategies. Finally, well-designed clinical trials will be essential to validate the therapeutic relevance of ASX in human pathologies, especially in inflammatory, metabolic, and degenerative diseases.

## Data Availability

No new data were created.

## Abbreviations

The following abbreviations are used in this manuscript:

ASC	Adipose-Derived Stem Cells
ASX	Astaxanthin
ROS	Reactive Oxygen Species
PI3K	Phosphoinositide-3-Kinase
Akt	Protein Kinase B
Nrf2	Nuclear Factor Erythroid 2-Related Factor 2
NF-κB	Nuclear Factor Kappa-Light-Chain-Enhancer of Activated B Cells
TNF-α	Tumor Necrosis Factor-α
IL-6	Interleukin-6
iNOS	Inducible Nitric Oxide Synthase
CO_2_	Carbon Dioxide
H-aggregates	Hypsochromic Aggregates
J-aggregates	Bathochromic Aggregates
GPX	Glutathione Peroxidase
SOD	Superoxide Dismutase
CAT	Catalase
HO-1 Heme	Oxygenase 1
NQO1	NAD(P)H Quinone Dehydrogenase 1
GST	Glutathione S-Transferase
GCLC	Glutamate-Cysteine Ligase Catalytic Subunit
GSR	Glutathione Reductase
TP53	Tumor Protein p53
PUMA	p53 Upregulated Modulator of Apoptosis
BIM	Bcl-2 Interacting Mediator of Cell Death
NOXA	Phorbol-12-Myristate-13-Acetate-Induced Protein 1
BCL-2	B-cell Lymphoma 2
PRRs	Pattern Recognition Receptors
PAMPs	Pathogen-Associated Molecular Patterns
DAMPs	Damage-Associated Molecular Patterns
LOX-1	Lectin-Like Oxidized LDL Receptor-1
CD36	Cluster of Differentiation 36
MMPs	Matrix Metalloproteinases
RTK	Receptor Tyrosine Kinase
GPCRs	G Protein-Coupled Receptors
IRS-1/2	Insulin Receptor Substrate 1/2
GLUT4	Glucose Transporter Type 4
PPARγ	Peroxisome Proliferator-Activated Receptor Gamma
TIF2	Transcription Intermediary Factor 2
SRC-1	Steroid Receptor Coactivator-1
ETC	Electron Transport Chain
ER	Endoplasmic Reticulum
ECM	Extracellular Matrix
MAPK	Mitogen-Activated Protein Kinase
TGF-β	Transforming Growth Factor Beta
CXCL9	C-X-C Motif Chemokine Ligand 9
CXCR3	C-X-C Motif Chemokine Receptor 3
miRNA34	MicroRNA 34
